# Interpreting scratch assays using pair density dynamics and approximate Bayesian computation

**DOI:** 10.1098/rsob.140097

**Published:** 2014-09-10

**Authors:** Stuart T. Johnston, Matthew J. Simpson, D. L. Sean McElwain, Benjamin J. Binder, Joshua V. Ross

**Affiliations:** 1 School of Mathematical Sciences, Queensland University of Technology, Brisbane, Australia; 2 Institute of Health and Biomedical Innovation, Queensland University of Technology, Brisbane, Australia; 3 School of Mathematical Sciences, University of Adelaide, Adelaide, Australia

**Keywords:** cell motility, cell proliferation, scratch assay, approximate Bayesian computation, cancer, pair correlation

## Abstract

Quantifying the impact of biochemical compounds on collective cell spreading is an essential element of drug design, with various applications including developing treatments for chronic wounds and cancer. Scratch assays are a technically simple and inexpensive method used to study collective cell spreading; however, most previous interpretations of scratch assays are qualitative and do not provide estimates of the cell diffusivity, *D*, or the cell proliferation rate, *λ*. Estimating *D* and *λ* is important for investigating the efficacy of a potential treatment and provides insight into the mechanism through which the potential treatment acts. While a few methods for estimating *D* and *λ* have been proposed, these previous methods lead to point estimates of *D* and *λ*, and provide no insight into the uncertainty in these estimates. Here, we compare various types of information that can be extracted from images of a scratch assay, and quantify *D* and *λ* using discrete computational simulations and approximate Bayesian computation. We show that it is possible to robustly recover estimates of *D* and *λ* from synthetic data, as well as a new set of experimental data. For the first time, our approach also provides a method to estimate the uncertainty in our estimates of *D* and *λ*. We anticipate that our approach can be generalized to deal with more realistic experimental scenarios in which we are interested in estimating *D* and *λ*, as well as additional relevant parameters such as the strength of cell-to-cell adhesion or the strength of cell-to-substrate adhesion.

## 2. Introduction

Scratch assays, otherwise known as scrape or wound healing assays [1,2], are a common experimental method used to study collective cell spreading. Cells are grown to confluence on a culture plate, after which an artificial gap is created in the monolayer with a fine-tipped instrument [1]. Microscopic images of the cell front moving into the vacated area are captured over approximately 12–24 h [3–6]. Scratch assays are often used to evaluate the impact of biochemical compounds on cell migration and proliferation [7–10]. For example, scratch assays have been used to study wound healing treatments [9,11], compounds that promote metastasis [7] and chemotherapeutic drugs [8]. Unfortunately, the majority of these evaluations are qualitative [5,11], or focus on measurements that do not distinguish between the roles of cell diffusivity and cell proliferation [7–9,12,13]. Quantitative comparisons between control assays and assays where a treatment has been applied are critical to providing information about the efficacy of a treatment. There is therefore considerable interest in the development of robust approaches that recover estimates of the cell diffusivity *D* and cell proliferation rate *λ*, as these parameters provide important information about the effectiveness and the mechanism of action of a putative treatment.

Previous quantitative interpretations of scratch assays have considered a variety of experimental measurements, including counting cell numbers to construct detailed cell density profiles [14–17], estimating the position of the leading edge of the spreading population [12,18,19] and recording detailed individual cell trajectories [14,16]. In some cases, these measurements have been compared with the results of a mathematical model to produce point estimates of *D* and *λ* [20,21]. Presently, it is unclear whether some of these experimental measurements lead to improved estimates of *D* and *λ* relative to other experimental measurements, and it remains unclear whether an optimal experimental measurement from a scratch assay can be identified. To the best of our knowledge, pair density information and pair correlation functions [22,23] have not been previously considered as a means of estimating *D* and *λ* from a scratch assay. Unlike previous quantitative interpretations, the data required to calculate the pair correlation function from a scratch assay is straightforward to obtain since it can be calculated simply by inspecting images of the assay at several time points without detailed cell labelling techniques or real-time tracking of individual cells. Calculating the pair correlation function from experimental images incorporates information about both the counts of pair distances and the number of cells in the image. This kind of information can also be easily extracted from discrete, individual-based random walk simulations incorporating random cell movement (governed by the cell diffusivity *D*) and cell proliferation (governed by the cell proliferation rate *λ*).

Typically, *D* and *λ* are estimated by minimizing a measure of the difference between some experimental measure and a prediction of a mathematical model, giving rise to point estimates of *D* and *λ* [12,14,19,21]. However, any information about the uncertainty of the recovered parameters is ignored by this standard approach. Understanding and quantifying the uncertainty in our estimates are important since previously reported estimates of *D* vary widely [21], and so it is insightful to employ parameter estimation techniques that provide more information than traditional approaches. Approximate Bayesian computation (ABC) generates a parameter distribution that contains this information, and hence provides more insight into the recovered parameters [24–26]. The use of ABC algorithms in spatio-temporal problems is relatively novel and has not been considered in the context of a scratch assay.

As far as we are aware, the application of ABC techniques to interpret scratch assays using random walk computer simulations has not been attempted previously. Therefore, in this work we focus on a relatively straightforward experimental system by working with 3T3 fibroblast cells, which are widely assumed to undergo migration and proliferation without significant cell-to-cell or cell-to-substrate adhesion effects [14,16,17]. This simplification allows us to focus on the estimation of two parameters, *D* and *λ*. Of course, if the technique described in this work were to be applied to other cell types where other mechanisms (such as cell-to-cell adhesion, cell-to-substrate adhesion or other mechanical effects) were present, a more detailed random walk framework with additional parameters would be required. For example, Khain *et al.* [27] describe such an extension whereby individual motility events in the random walk simulation are affected by adhesion, and this is incorporated into the computer simulations through the use of an additional parameter. Other extensions are also possible, such as the incorporation of mechanical forces [28–30]. While this work does not incorporate these additional details, we anticipate that the general framework presented here for the simpler random walk simulations with just two parameters could be extended to deal with these further details in future applications.

Here, we interpret new experimental images from a scratch assay using discrete random walk simulations, pair correlation functions and ABC. In §3, we describe the experimental procedure, present a random walk simulation framework that approximates the behaviour of cells in a scratch assay [31] and describe the process of comparing the simulation predictions with experimental data. We note that the random walk model is applied by performing repeated stochastic computational simulations, and henceforth we refer to our random walk model as a computational simulation. In §4, we present the results from an ABC algorithm applied to synthetically generated data, and compare our ability to estimate *D* and *λ* using various pieces of information from the images of the synthetic scratch assay. We show that combining estimates of the pair correlation function and the number of cells in the image allows us to robustly estimate *D* and *λ*. Applying the same technique to new experimental data, we recover estimates of *D* and *λ* that are well defined and consistent with previous point estimates [17]; however, we also present information about the uncertainty in our parameter estimates that has not been presented previously. In §5, we discuss our results and suggest directions for future study.

## 3. Material and methods

### 3.1. Experimental method

The details of the experimental method have been presented previously [32]. Briefly, murine fibroblast 3T3 cells [33] were grown in T175 cm^2^ tissue culture flasks. One microlitre of cell suspension was carefully inserted into the well of a tissue culture plate to ensure that cells were approximately evenly distributed. The tissue culture plate was placed in a humidified incubator at 37°C and 5% CO_2_ until the population became confluent. A scratch was made in the population using a P1000 pipette tip (Lab Advantage, Australia). Images of the spreading population were recorded using a Leica AF6000 automated microscope every 5 min for 24 h.

### 3.2. Computational simulation

We consider a discrete random walk incorporating motility and proliferation mechanisms on a two-dimensional square lattice with lattice spacing Δ, where each lattice site may be occupied by, at most, one agent [31,34]. At time *t*, the lattice contains *N*(*t*) agents, which have the ability to move and proliferate, with probability *P*_m_ ∈ [0,1] and *P*_p_ ∈ [0,1], respectively, during each timestep of fixed duration *τ*. Invoking the standard assumption that *P*_m_ and *P*_p_ are constant, the parameters in the computational simulation are related to *D* and *λ* by [31]3.1
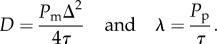
Using these relationships, we can treat the parameters in the simulation, *P*_m_ and *P*_p_, as interchangeable with *D* and *λ*, respectively.

During each timestep, *N*(*t*) agents are selected with replacement, at random, one at a time [35], and given a chance to move [31]. Once selected, an agent at (*x,y*) randomly chooses and attempts to move to either (*x* ± Δ,*y*) or (*x,y* ± Δ). Potential motility events are successful provided that the target site is vacant; otherwise, the event is aborted. After *N*(*t*) motile events have been attempted, an additional *N*(*t*) agents are selected with replacement, at random, one at a time and are given the opportunity to proliferate. A proliferative agent at (*x,y*) attempts to place a daughter agent at (*x* ± Δ,*y*) or (*x,y* ± Δ). Attempted proliferation events can only be successful if the target site is unoccupied; otherwise, the event is aborted. We note that our random walk simulation is an idealization in which it is always theoretically possible that an agent in the simulations could occasionally proliferate twice in quick succession, and we note that this is not biologically relevant. However, for parameter values relevant to our biological system (and many others), this feature is expected to have minimal impact. To see this, we note that the average time between motility events for an isolated agent is *τ*/*P*_m_, whereas the average time between proliferation events for an isolated agent is *τ*/*P*_p_. Therefore, for our simulations to be biologically realistic, we expect the quantity *τ*/*P*_m_ to be in the order of 10–20 min [27], but the quantity *τ*/*P*_p_ to be in the order of approximately 24–48 h [19]. We will make a comment on these details in §4.2 when we interpret our results.

**Figure 1.**
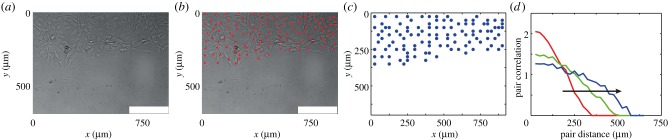
(*a*) Typical experimental image obtained from a scratch assay performed using 3T3 fibroblast cells. (*b*) Identification of the position of the cells. Scale bar corresponds to 250 μm. (*c*) Position of cells mapped to a square lattice, where the lattice size is equal to the cell diameter, Δ = 25 μm. (*d*) Pair correlation function *q*(*i*), obtained from experimental images at time *t* = 4 h (red), *t* = 8 h (green), *t* = 12 h (blue). Arrow indicates direction of increasing time. See §3.3 and the electronic supplementary material for details about the calculation and interpretation of *q*(*i*).

We choose the geometry of our simulation to mimic the scratch assay presented in figure 1*a,b*. The average cell diameter is approximately 25 μm [17], giving Δ = 25 μm. The simulation domain (an *X* by *Y* lattice, presented in figure 1*c*) corresponds with the size of the experimental images. The image in figure 1*a* is approximately 900 μm wide and 675 μm high, corresponding to *X* = 36 and *Y* = 27. We apply symmetry (zero flux) boundary conditions along the boundaries at *x* = 0, *x* = *X*Δ, *y* = 0 and *y* = *Y*Δ. To initiate the computational simulation, we place *N*(0) agents, at random, ensuring that no two agents occupy the same site, in the region for *y* ≤ *Y*_0_Δ. We estimate *N*(0) by counting the number of cells present at *t* = 0 in the experimental images. We note that *N*(*t*) depends on time *t*, but we refer to this quantity as *N* from this point for notational convenience.

### 3.3. Pair correlation functions

There is a significant amount of information available in an experimental image of a scratch assay. For example, cell density profiles [14,17], individual cell trajectories [14] and the position of the leading edge of the spreading cell front [12,19] have all been estimated from experimental images, and used to provide point estimates of *D* and *λ*. Here, we consider estimating the pair correlation function [22] as an experimental measurement, henceforth referred to as a summary statistic. Summary statistics are lower-dimensional summaries of data that provide tractable comparisons between sets of data [24]. Since summary statistics merely summarize a dataset, it is important to examine whether a particular summary statistic is sufficient; that is, a statistic that contains all information about the parameters available from the experiment.

To calculate the pair correlation function, we consider a dataset corresponding to a square lattice of dimensions *X* by *Y*, where each lattice site can be occupied by, at most, one agent. Each lattice site has an index (*x,y*), where 1 ≤ *x* ≤ *X*, 1 ≤ *y* ≤ *Y*. All occupied lattice sites at time *t*, (*x_j_*,*y_j_*), are uniquely indexed by *j* = 1, … , *N*. The number of occupied lattice pairs for each pair distance *i* = 1, … , *Y* − 1 is then given by3.2
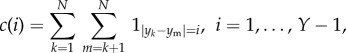
where 1*_a_* is the indicator function, which is equal to one if *a* is true and is equal to zero otherwise. We have oriented our lattice such that the *x* direction is parallel to the direction of the initial scratch and the *y* direction is perpendicular to the direction of the initial scratch. Previous analysis [22] indicates that there is more information in the *y* direction for this kind of scratch assay, and so we focus on counting the pairs of agents in the *y* direction from this point onward.

Binder & Simpson [22] demonstrated that it was possible to normalize equation (3.2) to produce a pair correlation function which accounted for volume exclusion and crowding effects, and here we use the same approach. Binder & Simpson's [22] normalization term describes the expected number of pairs of occupied lattice sites, for each pair distance *i*, in a randomly distributed population without any spatial correlation. The normalization term is given by3.3
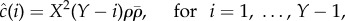
where *ρ* = *N*/(*XY*), 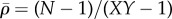 and *N* is the number of occupied lattice sites. Therefore, the pair correlation function is given by3.4
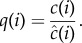
We note that *q*(*i*) is a non-dimensional measure of the abundance of pairs of objects relative to a uniformly distributed population, whereas *c*(*i*) is a dimensional measure of the number of pairs. Intuitively, we expect that measurements relating to pair density information could provide important information about the rates of cell motility and cell proliferation since proliferation events produce pairs separated by a short distance, whereas motility events act to increase the distance between cell pairs. However, without any quantitative comparisons, it is unclear whether there is any advantage in using *q*(*i*) or *c*(*i*) to recover estimates of *D* and *λ*.

To compare our experimental data with the predictions from our computational simulation, we map the positions of cells in the experimental images onto the same lattice used in the simulation (electronic supplementary material). We then calculate the pair correlation function *q*(*i*), for both the experimental images and the images produced by the computational simulation, using the method outlined by Binder & Simpson [22]. Values of *q*(*i*) greater than unity indicate that the distribution of cells or agents is such that we are more likely to find a pair of cells or agents separated by a distance *i* than in a spatially uniform distribution. Similarly, values of *q*(*i*) less than unity indicate that the distribution of cells or agents is such that we are less likely to find a pair of cells or agents separated by a distance *i* than in a spatially uniform distribution. If we find that *q*(*i*) is approximately unity for all pair distances, the domain is populated uniformly at random [22]. Calculating *q*(*i*) requires information about the counts of pair distances *c*(*i*), and the number of cells or agents *N*. To calculate the pair correlation function, we normalize the pair distance counts by the density, which depends on *N*. Therefore, we expect some information regarding *N* will be lost when considering the pair correlation function only as a summary statistic. However, since the pair correlation function has been used previously to analyse *in vitro* cell biology assays [22,23], it is relevant for us to examine whether there is sufficient information in the pair correlation function to robustly recover estimates of *D* and *λ* using an ABC framework. For completeness, in §4.1, we also examine and compare results generated by considering other potential summary statistics to ensure that we use the most appropriate information in our parameter estimation.

The process of mapping cells from a continuous image onto a lattice can involve some discretization error, which we investigated in detail recently [23]. This previous study explored the impact of using differently sized lattices to discretize similar experimental images, and we computed the pair correlation function for variously sized lattices, and this showed that the pair correlation function was insensitive to the size of the lattice provided that the lattice spacing was at least as small as the average cell diameter [23].

### 3.4. Approximate Bayesian computation

ABC is a useful method for computing posterior distributions of unknown model parameters in situations where the likelihood function is intractable [36]. ABC algorithms consider parameter values that generate model predictions that attempt to match observed experimental data [24–26,36,37]. To approximate the posterior distribution *f*(*θ*|*β*), we consider a prior distribution, *π*(*θ*), and a simulation that provides a summary statistic based on a parameter set, *θ*. We note that *β* represents the experimental data and define *S*(*β*) as the corresponding summary statistic. Making minimal assumptions, we consider a uniform prior, *P*_m_ ∈ [0,1], *P*_p_ ∈ [0,1], to generate parameter values and corresponding simulations. Given that the time scale of cell proliferation is typically much larger than the time scale of cell motility, 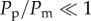 [31], we anticipate that a significant region of the parameter space will not produce realizations that match the experimental data. To reduce the computation time, we therefore implement the ABC Markov chain Monte Carlo algorithm (ABC-MCMC) [24,36], an ABC algorithm that evolves based on previously successful parameter values (electronic supplementary material).

## 4. Results

### 4.1. Synthetic data

**Figure 2.**
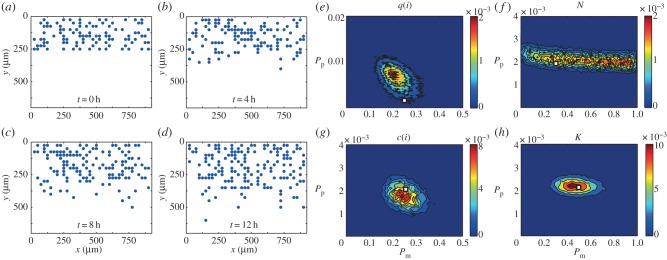
(*a–d*) Typical evolution of the discrete simulation described in §3.2, generated with (*P*_m_, *P*_p_) = (0.25, 2 × 10*^−^*^3^), presented at time (*a*) *t* = 0 h, (*b*) *t* = 4 h, (*c*) *t* = 8 h and (*d*) *t* = 12 h. (*e–h*) Calculated posterior distribution obtained from a summary statistic generated with (*P*_m_, *P*_p_) = (0.25, 2 × 10*^−^*^3^) using (*e*) the pair correlation function *q*(*i*), (*f*) the number of cells *N*, (*g*) the counts of pair distances *c*(*i*) and (*h*) *K*, the combination of *q*(*i*) and *N* as the summary statistic. The details of the process to obtain the distributions are given in the electronic supplementary material. In brief, 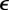 is the maximum difference between the summary statistics for *θ* to be accepted, *Γ* is the width of the distribution of potential step sizes in the ABC-MCMC algorithm and *M* is the total number of steps attempted. Parameter values used were (*e*) 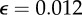, (*f*) 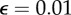, (*g*) 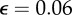 and (*h*) 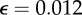. 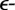
values were chosen such that the posterior distribution did not significantly change if 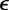 was reduced (electronic supplementary material). For all simulations *τ* = 1/24 h, *Γ* = (10*^−^*^1^, 10*^−^*^3^), *M* = 10^6^, *N*(0) = 100, *Y*_0_ = 10. Red areas indicate high relative frequency while blue areas indicate low relative frequency. All simulation data are insensitive to *τ*. The white squares represent the parameter values used to generate the synthetic data.

To examine the robustness of our method and the validity of using the pair correlation function as a summary statistic, we first attempt to recover parameter values from data generated synthetically. We choose a biologically relevant parameter set (*P*_m_, *P*_p_) = (0.25, 2 × 10 *^−^* ^3^), which with Δ = 25 μm and *τ* = 1/24 h corresponds to (*D*, *λ*) = (937.5 μm^2^ h^−1^, 4.8 × 10 *^−^* ^2^ h^−1^). We perform a single realization of the simulation with these parameters and calculate the relevant summary statistics at time *t* = 4, 8, 12 h. The average distance between the summary statistic for the synthetic data and the simulation prediction at *t* = 4, 8, 12 h is calculated and compared to either accept or reject potential parameter values to estimate the posterior distribution (electronic supplementary material). The evolution of the computational simulation is presented in figure 2*a–d*. We apply the ABC-MCMC algorithm (electronic supplementary material) and present the resulting posterior distribution for the pair correlation function *q*(*i*), in figure 2*e*. If the pair correlation function were a close-to-sufficient summary statistic, we would observe a well-defined posterior distribution centred at (*P*_m_, *P*_p_) = (0.25, 2 × 10*^−^*^3^), with a narrow spread about the mean in the distributions of *P*_m_ and *P*_p_. Instead, we observe that the posterior distribution is centred at (*P*_m_, *P*_p_) ≈ (0.22, 6.7 × 10*^−^*^3^), with significant spread. These observations suggest that additional information ought to be incorporated into our ABC algorithm. We note that identically prepared simulations using the same values of Δ and *τ*, but different values of *P*_m_ and *P*_p_, can occasionally produce similar or indistinguishable summary statistics. This is due to the fact that our random walk computer simulations are stochastic. For this reason, we feel it is more appropriate to interpret our experimental results using a probabilistic ABC approach, leading to a distribution of *D* and *λ*, rather than using a more traditional approach and arriving at point estimates of *D* and *λ*.

We now attempt to refine our estimates of *P*_m_ and *P*_p_ by examining the posterior distributions obtained by considering the number of cells *N*, and the pre-normalized counts of the pair distances *c*(*i*), as summary statistics in figure 2*f* and *g*, respectively. Intuitively, we expect that *N* may provide some information about *P*_p_ but less information about *P*_m_. Indeed, the posterior distribution in figure 2*f* suggests that all values in the range *P*_m_ ∈ [0,1] are potentially acceptable and there is little correlation between *P*_m_ and *P*_p_. The counts of pair distances correspond to the pre-normalized pair correlation functions. Since information regarding *P*_m_ and *P*_p_ may be lost in the normalization that converts *c*(*i*) into *q*(*i*), we anticipate that *c*(*i*) could be a more relevant summary statistic than *q*(*i*). We observe in figure 2*g* that *c*(*i*) is indeed an excellent summary statistic as the calculated posterior distribution is centred on (*P*_m_, *P*_p_) ≈ (0.25, 2 × 10*^−^*^3^).

The final summary statistic we consider is the average of *q*(*i*) and *N*, *K* = {*d*[*q*(*i*)] + *d*[*N*]/2}, where *d*[*L*] is a measure of the difference between two datasets using an arbitrary summary statistic *L* (electronic supplementary material). Therefore, the summary statistic *K* includes information about the number of cells or agents lost due to the formulation of the pair correlation function, *q*(*i*). We present the posterior distribution calculated using *K* in figure 2*h* and we find that the distribution is similarly centred on (*P*_m_, *P*_p_) ≈ (0.25, 2 × 10*^−^*^3^). Owing to the explicit inclusion of *N* in the summary statistic, we observe a reduction in the spread of the posterior distribution in the *P*_p_ direction, compared to figure 2*g*, while maintaining a similar spread in the *P*_m_ direction. While in theory we could continue to incorporate additional information in our summary statistic to obtain a further refined posterior distribution, there is an important computational trade-off between the more complicated summary statistic and the improvement in the posterior distribution [38].

**Figure 3.**
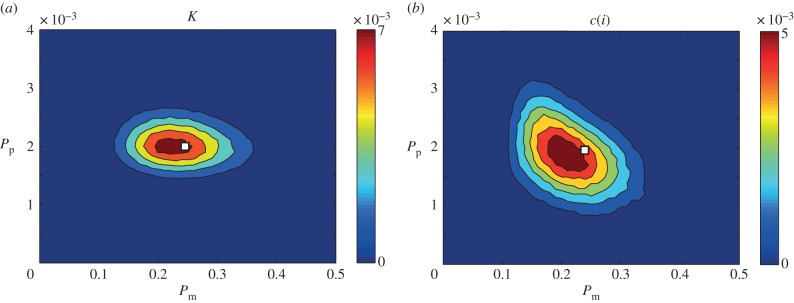
Averaged posterior distributions calculated using summary statistics obtained simulations as prepared in figure 2 for (*a*) *K*, a combination of the pair correlation function *q*(*i*) and the number of cells *N*, and (*b*) the counts of pair distances *c*(*i*), as a summary statistic. The details of the process to obtain the distributions are given in the electronic supplementary material. Parameter values used were (*a*) 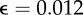 and (*b*) 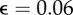. For all simulations *τ* = 1/24 h, *Γ* = (10*^−^*^1^, 10*^−^*^3^), *M* = 10^6^, *N*(0) = 100, *Y*_0_ = 10. The white squares represent the parameter values used to generate the synthetic data. For further details about the parameters, see figure 2.

As both the *c*(*i*) and *K* summary statistics lead to reasonable posterior distributions, we now compare them by repeating the ABC-MCMC algorithm on 10 sets of identically prepared simulation data (that is, simulation data generated using the same parameter values, initial and boundary conditions) and investigate the average of the 10 resulting posterior distributions, shown in figure 3. We observe that the distribution in figure 3*a* using *K* as the summary statistic has a significantly smaller spread than in figure 3*b*, which used *c*(*i*) as the summary statistic. To quantitatively compare the posterior distributions, we calculate the Kullback–Leibler divergence [39], which is a measure of the information gained from moving from the prior to the posterior distribution, and is defined as4.1
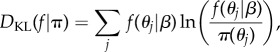
where the index *j* accounts for all possible discretized parameter pairs. A larger *D*_KL_(*f|*π) value implies that more information is obtained when moving from the prior to the posterior distribution [39]. We discretize our posterior distribution onto a lattice with 10^2^ equally spaced values of *P*_m_ in the interval *P*_m_ ∈ [0,1] and 10^4^ equally spaced values of *P*_p_ in the interval *P*_p_ ∈ [0,1], and count the number of successful observations for each parameter combination, and use this information to calculate *D*_KL_(*f|*π). We find that *D*_KL_(*f|*π) is higher for the posterior distribution calculated using *K* rather than *c*(*i*): *D*_KL_(*f|*π) = 7.98 and *D*_KL_(*f|*π) = 7.61, respectively. For perspective, the *D*_KL_(*f|*π) values for *q*(*i*) and *N* were 6.32 and 6.93, respectively. Therefore, a difference in *D*_KL_(*f|*π) of approximately 0.3 is relevant. Guided by this information, we will interpret our experimental data using *K* as the summary statistic.

### 4.2. Experimental data

**Figure 4.**
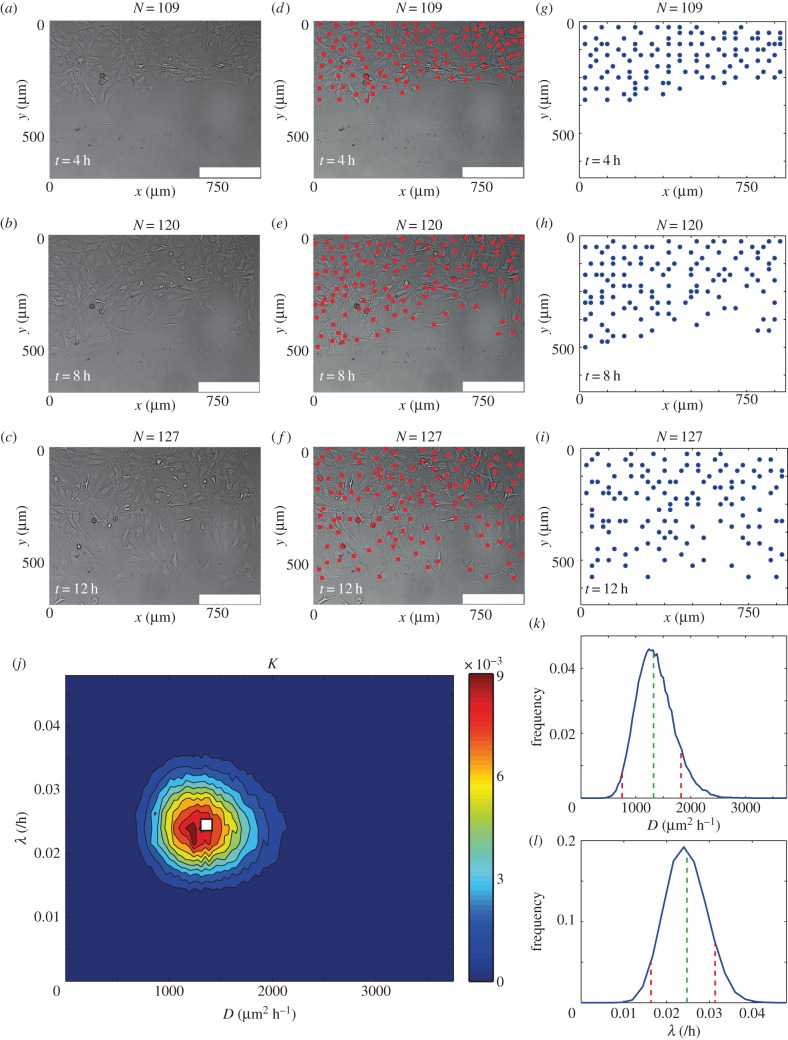
(*a*–*c*) Typical experimental images of a scratch assay at (*a*) *t* = 4 h, (*b*) *t* = 8 h and (*c*) *t* = 12 h. (*d–f*) Experimental images of a scratch assay with the position of cells indicated (red squares) at (*d*) *t* = 4 h, (*e*) *t* = 8 h and (*f*) *t* = 12 h. (*g*–*i*) The position of cells when mapped onto a lattice at (*g*) *t* = 4 h, (*h*) *t* = 8 h, (*i*) *t* = 12 h. (*j*) Posterior distribution calculated using *K*, which is the average of *q*(*i*) and *N*, as a summary statistic and 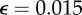. The details of the process to obtain the distribution are given in the electronic supplementary material. In brief, 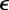 is the maximum difference between the summary statistics for *θ* to be accepted, *Γ* is the width of the distribution of potential step sizes in the ABC-MCMC algorithm and *M* is the total number of steps attempted. For all simulations, *τ* = 1/24 h, *Γ* = (10*^−^*^1^, 10*^−^*^3^), *M* = 10^6^, *N*(0) = 102, *Y*_0_ = 10. Red areas indicate high relative frequency while blue areas indicate low relative frequency. 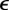 was chosen such that the posterior distribution did not significantly change if 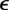 was reduced (electronic supplementary material). The white square represents the mean parameter values, (*D*, *λ*) ≈ (1350 μm^2^ h^−1^, 2.5 × 10*^−^*^2^ h^−1^). All simulation data are insensitive to *τ*. (*k*) Posterior distribution of *λ* obtained by averaging over *D* with the mean, *λ* = 2.5 × 10*^−^*^2^ h^−1^, (dashed green) and 90% credible interval, (1.7 × 10*^−^*^2^−3.1 × 10*^−^*^2^)/h (dashed red) superimposed. (*l*) Posterior distribution of *D* obtained by averaging over *λ* with the mean, *D* = 1350 μm^2^ h^−1^, (dashed green) and 90% credible interval, (675−1800) μm^2^ h^−1^ (dashed red), superimposed.

We obtained experimental data from a scratch assay, calculated *q*(*i*) and *c*(*i*), and counted *N*, at *t* = 4, 8, 12 h. The position of the cells in the experiments was mapped to a square lattice with Δ = 25 μm (electronic supplementary material). Figure 4*a–i* illustrates the process of mapping the cell positions to the lattice. We applied the ABC-MCMC algorithm (electronic supplementary material) to the experimental data using *K* as a summary statistic and the average distance between the summary statistic for the experimental data and the simulation prediction at *t* = 4, 8, 12 h to either reject or accept potential parameter values in the estimation of the posterior distribution, given in figure 4*j*. We observe that the resulting bivariate posterior distribution is well defined and contains a relatively narrow spread in both the *D* and *λ* directions. To provide further insight, we estimate the corresponding univariate distributions of *D* and *λ*, presented in figure 4*k–l*, by averaging the posterior distribution in each of the *λ* and *D* directions, respectively. Since the univariate posterior distributions do not appear to be significantly skewed, we choose to report the mean of the univariate posterior distributions as our estimate of *D* and *λ*. To provide quantitative insight into the uncertainty in our estimates, we calculate the 90% credible interval by finding the interval, symmetric about the mode, containing approximately 90% of the total area under the univariate distribution. The mean and 90% credible intervals are *D* ≈ 1350(675–1800) μm^2^ h^−1^ and *λ* ≈ 2.5 × 10*^−^*^2^(1.7 × 10*^−^*^2^–3.1 × 10*^−^*^2^) h^–1^. We note that our estimates of *D* and *λ* are consistent (within a factor of two) with previously reported point estimates [17]. However, unlike previous point estimates of *D* and *λ*, our approach provides a well-defined quantitative estimate of the uncertainty present in the parameter recovery. Furthermore, our approach does not require overly complicated and time-consuming experimental procedures such as tracking individual cells [14,16], constructing cell density profiles [14] or performing multiple sets of assays in which proliferation is artificially suppressed [17].

For our parameter estimates (*D* ≈ 1350 μm^2^ h^−1^ and *λ* ≈ 2.5 × 10*^−^*^2^ h^−1^) with Δ = 25 μm and *τ* = 1/24 h, equation (3.1) gives *P*_m_ = 0.36 and *P*_p_ = 0.00104. Therefore, the average time between motility events for an isolated agent *τ*/*P*_m_ is approximately 7 min, whereas the average time between proliferation events for an isolated agent *τ*/*P*_p_ is approximately 40 h. These quantities are biologically realistic since the time scale of cell motility is much shorter than the time scale of cell proliferation, and these quantities are consistent with previous estimates of the time scale of cell motility [27] and previous estimates of the time scale of cell proliferation [19]. While it is possible to impose additional conditions on our computational simulations, such as explicitly ensuring that no two proliferation events ever occur in rapid succession, we have avoided introducing such details to ensure that our computational simulations are consistent with previously reported algorithms [31].

## 5. Discussion and conclusion

Scratch assays are a technically simple and inexpensive method used to observe spreading cell fronts [1], which can be thought of as a simple representation of wound healing, malignant spreading or certain developmental processes. The impact of biochemical compounds on cell diffusivity and cell proliferation, vital to cancer and wound healing research, can, in principle, be measured using a scratch assay [7–10]. However, the majority of previous studies have reported qualitative data [5,11], which cannot separately identify *D* and *λ* or the impact of the potential treatment on *D* and *λ* [7–9,12]. While mathematical models have been used to obtain separate point estimates of *D* and *λ* [14,15,19,20], these previous studies have neglected to consider the uncertainty present in the parameter recovery process.

The work presented here addresses two common limitations of previous interpretations of scratch assays. First, our method provides quantitative estimates of *D* and *λ* by comparing images from a scratch assay with predictions from a lattice-based computational simulation of cell migration and proliferation. Second, to compare the experimental images with the simulation we implement an ABC-MCMC algorithm with an appropriate summary statistic to approximate the posterior distribution of *D* and *λ*. The posterior distribution contains vital information about the uncertainty and variability of the recovered parameters, information that is not present in previous interpretations of scratch assays. Using an ABC technique that quantifies this uncertainty will be useful for investigating the efficacy of putative drug treatments, which could be relevant for studying both wound healing [10] and cancer [8]. For example, a traditional approach of estimating *D* and *λ* could be used to provide point estimates of *D* and *λ* under control conditions, and compare these to separate point estimates of *D* and *λ* for an experiment in which the drug has been applied. Alternatively, our approach could be used to produce a distribution of *D* and *λ* under control conditions and compare these distributions to those obtained by analysing a set of equivalent experiments where the drug was applied. Comparing distributions of *D* and *λ* provides additional information that is not possible when comparing point estimates. For example, it allows us to assess our confidence in stating that one treatment is better than another. Furthermore, it will assist us in determining an appropriate number of experimental comparisons to ensure reliable assessment.

Our approach for estimating *D* and *λ* from a scratch assay provides more comprehensive information than a traditional method, which typically produces point estimates of *D* and *λ* only. However, one of the limitations of our approach is that it relies upon obtaining highly resolved images of the scratch assay such that the location of each cell in the image can be determined, and we acknowledge that this could be non-trivial in some situations. Although we have achieved this here using non-labelled cells, another approach might be to use some kind of nuclear stain to help identify the location of individual cells in the population [17].

To investigate the validity of applying ABC to spatio-temporal experiments such as scratch assays, we initially attempted to recover estimates of *P*_m_ and *P*_p_ from synthetic data generated using our computational simulation with pre-specified values of *P*_m_ and *P*_p_. By comparing different summary statistics, we found that using a weighted average of the pair correlation function *q*(*i*) and the number of cells or agents present *N* provided a simple yet insightful summary statistic. After confirming the validity of our approach using synthetic data, we applied the same approach to a new experimental dataset. Our posterior distribution of *D* and *λ* allowed us to estimate (*D*, *λ*) ≈ (1350 μm^2^ h^−1^, 2.5 × 10*^−^*^2^ h^−1^), which was consistent with previously reported estimates [17]. However, unlike previous point estimates, we also obtained information about the uncertainty present in the parameter recovery. The posterior distribution allowed us to estimate credible intervals for both *D* = (675−1800) μm^2^ h^−1^ and *λ* = (1.7 × 10*^−^*^2^−3.1 × 10*^−^*^2^) h^–1^ very simply using a single experimental dataset.

Our approach of interpreting scratch assays using ABC together with a combination of the pair correlation function and the number of cells present in the experimental images can be extended in several ways. For example, in this work we have only considered experimental data where cell-to-cell adhesion is negligible [33]. However, many cell types, such as glioma [27] and melanoma cells [40], exhibit significant cell-to-cell adhesion. An extension of the computational simulation framework presented here, such as the one presented by Khain *et al.* [27], could be employed to analyse scratch assays conducted with adhesive cells. Khain's random walk model includes an additional dimensionless parameter, 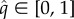, describing the strength of cell-to-cell adhesion, and it would be interesting to investigate whether there is sufficient information present in images from a scratch assay using adhesive cells to robustly recover estimates of Khain's three model parameters, *D*, *λ* and 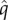. Furthermore, other types of mathematical model could be considered, with more detailed descriptions of cell migration and proliferation [30], other more detailed mechanisms of cell-to-cell interaction [41,42] or different types of mechanical interactions [28]. However, since the application of ABC techniques to interpret scratch assay data has not been previously attempted, this study focused on a relatively straightforward experimental system that could be interpreted with a model relying on just two parameters. Of course, further extensions are possible and these include applying three-dimensional random walk simulations to describe three-dimensional assays [43–45]. Alternatively, we could investigate the influence of the assumption of memoryless proliferation, particularly for applications where a large proliferation rate is relevant. Another possible extension of our present study is to explore the limitations of using a lattice-based random walk model. This could be achieved by repeating the ABC analysis using a lattice-free random walk [46,47], and comparing the estimates of *D* and *λ* in the lattice-based and lattice-free frameworks. While this comparison is, in principle, possible to carry out, we note that ABC techniques rely on repeated simulations of the random walk, and that lattice-free models of collective cell behaviour with crowding effects are significantly more computationally demanding than lattice-based models. Therefore, we leave the extension of applying ABC techniques to a lattice-free model for future analysis.

## Supplementary Material

Supplementary Material

## Data accessibility

The dataset supporting the results of this article is included within the article.
