# Supplementary material for: Interpreting scratch assays using pair density dynamics and approximate Bayesian computation
Source: Open Biol. 2014 Sep 10;4(9):140097. doi: 10.1098/rsob.140097 (PMC4185435; doi:10.1098/rsob.140097)
Supplement: Supplementary Material [file rsob140097supp1.pdf]

Interpreting scratch assays using pair density dynamics and approximate Bayesian computation

Stuart T Johnston[1,2] *Matthew J Simpson[1,2] DL Sean McElwain[1,2]
Benjamin J Binder[3] Joshua V Ross[3]

Keywords: cell motility; cell proliferation; scratch assay; approximate Bayesian computation; cancer; spatial correlation.

1. School of Mathematical Sciences, Queensland University of Technology, Brisbane, Australia.
*matthew.simpson@qut.edu.au.
2. Institute of Health and Biomedical Innovation, Queensland University of Technology, Brisbane, Australia.
3. School of Mathematical Sciences, University of Adelaide, Adelaide, Australia

1 Supplementary Material

1.1 Lattice mapping

To map the positions of cells from the experimental image, where cell position is a continuous variable, to a discrete lattice we first calculate the position of each cell. We then define a mapping from cell position (x_c, y_c) to lattice site (x_L, y_L) through the relationship

$$x_L = \left\lceil \frac{x_c}{\Delta} \right\rceil, \qquad y_L = \left\lceil \frac{y_c}{\Delta} \right\rceil, \tag{1}$$

where $\lceil x \rceil$ denotes the ceiling function.

1.2 ABC Algorithm

Marjoram *et al.* [24] provide a full description of this approach and here we only give a brief outline of the algorithm used in our study.

R1 If at θ step to θ' according to a transition kernel $w(\theta \to \theta')$.

R2 Simulate β' from the model using θ' and calculate the summary statistic $S(\beta')$.

R3 Calculate the distance $||S(\beta) - S(\beta')||$, using an appropriate distance measure.

R4 If $||S(\beta) - S(\beta')|| > \epsilon$ reject θ' and return to **R1**.

R5 Calculate

$$h = \min\left(1, \frac{\pi(\theta')w(\theta' \to \theta)}{\pi(\theta)w(\theta \to \theta')}\right).$$

R6 Accept θ' with probability h.

R7 Return to **R1** until M steps have been attempted.

Initially, we sample θ randomly from the prior distribution, until the corresponding summary statistic is sufficiently close to the experimental summary statistic. We define the transition kernel that proposes θ' values as a bivariate uniform distribution, so that $\theta' \in \theta \pm \Gamma$, where Γ defines the width of the uniform distribution. The transition kernel ensures that $P_m \in [0, 1]$, $P_p \in [0, 1]$ by truncating the bivariate uniform distribution at the boundaries of the parameter space, if necessary. To measure the differences between two summary statistics we define

$$d\left[S(\beta)\right] = ||S(\beta) - S(\beta')|| = \frac{1}{Q}\left[\sum_{i=1}^{Q}\left(\frac{S(\beta)_i - S(\beta')_i}{1 + S(\beta)_i}\right)^2\right]^{\frac{1}{2}}, \quad (2)$$

where $S(\beta)_i$ is the ith data point in $S(\beta)$ and Q is the number of data points in $S(\beta)$. We note that we take the average $d\left[S(\beta)\right]$ value when there are summary statistics taken at multiple time points.

1.3 Distribution Convergence

To examine whether the posterior distribution generated from our ABC algorithm approximates $f(\theta|\beta)$, we consider posterior distributions generated with different ϵ values. The posterior distributions are calculated using the same data sets and identically prepared simulations. If the change in the posterior distribution between ϵ values is insignificant then the estimated posterior distribution provides a close approximation to $f(\theta|\beta)$ [23]. In Figures 1(a)-(c) we present results using three different ϵ values that demonstrate that the posterior distribution approximately converges for $\epsilon = 0.012$. We observe that the distribution is centred at approximately the same position, with regard to P_m and P_p, and that the spread of the distribution in the P_m and P_p directions are consistent between Figures 1(b)-(c). We repeat this process in Figures 2-7 for all distributions presented in this work and demonstrate the values of ϵ chosen are appropriate.

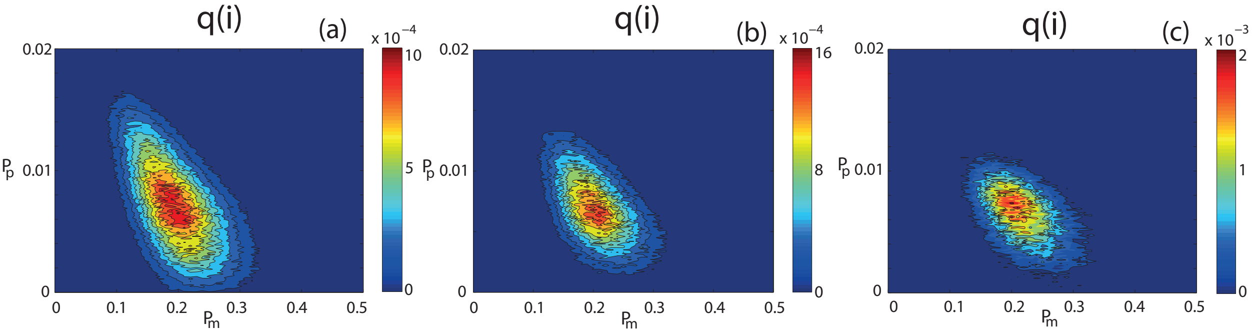

Figure 1: Convergence of the posterior distribution for a single synthetic data set for the summary statistic consisting of the pair correlation function $q(i)$. Synthetic data was generated with $P_m = 0.25$, $P_p = 2 \times 10^{-3}$. The maximum distance between summary statistics for θ to be accepted was (a) $\epsilon = 0.0135$, (b) $\epsilon = 0.012$, (c) $\epsilon = 0.011$. Red indicates high relative frequency while blue indicates low relative frequency.

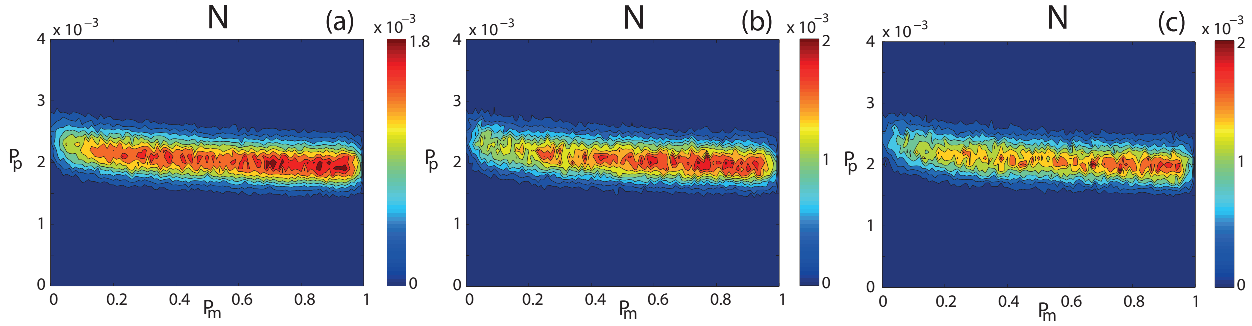

Figure 2: Convergence of the posterior distribution for a single synthetic data set for the summary statistic consisting of the number of cells N. Synthetic data was generated with $P_m = 0.25$, $P_p = 2 \times 10^{-3}$. The maximum distance between summary statistics for θ to be accepted was (a) $\epsilon = 0.012$, (b) $\epsilon = 0.012$, (c) $\epsilon = 0.01$. Red indicates high relative frequency while blue indicates low relative frequency.

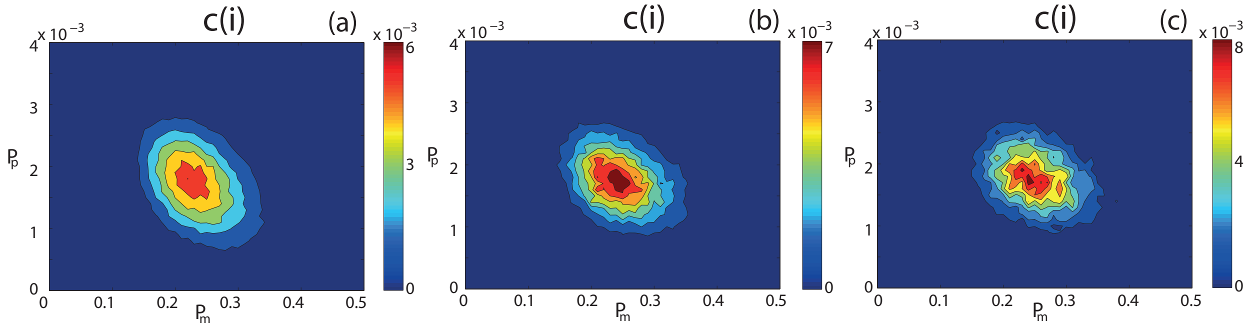

Figure 3: Convergence of the posterior distribution for a single synthetic data set for the summary statistic consisting of the counts of pair distances $c(i)$. Synthetic data was generated with $P_m = 0.25$, $P_p = 2 \times 10^{-3}$. The maximum distance between summary statistics for θ to be accepted was (a) $\epsilon = 0.075$, (b) $\epsilon = 0.065$, (c) $\epsilon = 0.06$. Red indicates high relative frequency while blue indicates low relative frequency.

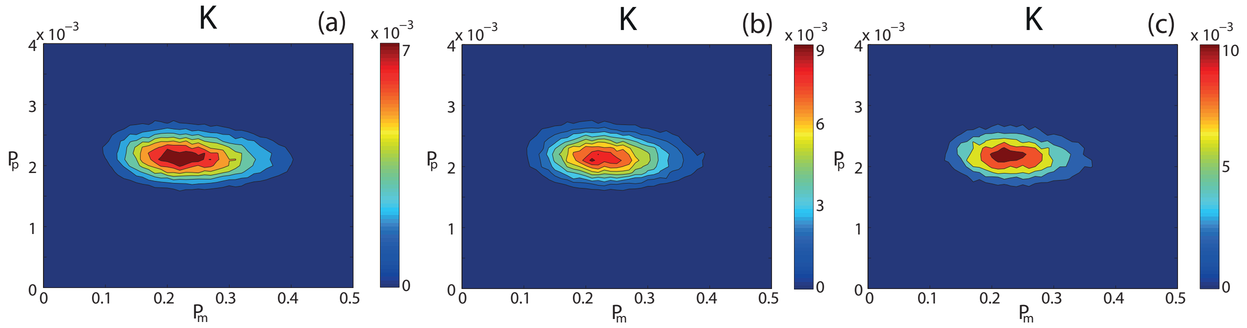

Figure 4: Convergence of the posterior distribution for a single synthetic data set for the K summary statistic, where $K = \{d[q(i)] + d[N]\}/2$. Synthetic data was generated with $P_m = 0.25$, $P_p = 2 \times 10^{-3}$. The maximum distance between summary statistics for θ to be accepted was (a) $\epsilon = 0.015$, (b) $\epsilon = 0.0135$, (c) $\epsilon = 0.012$. Red indicates high relative frequency while blue indicates low relative frequency.

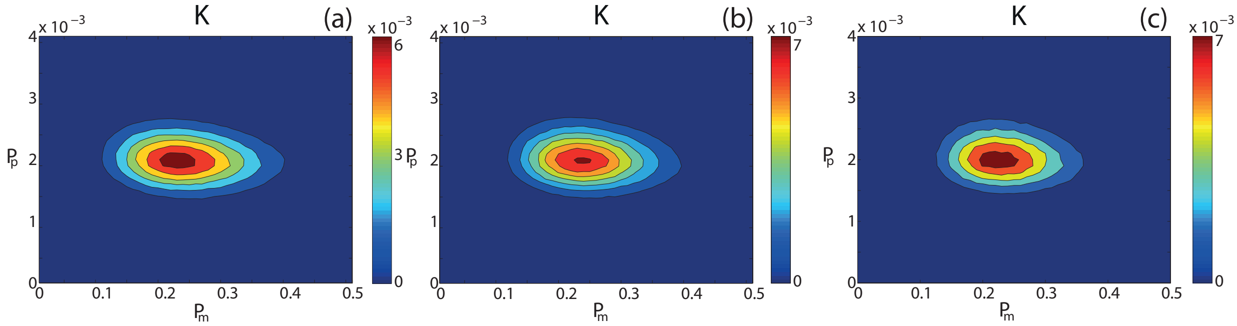

Figure 5: Convergence of the averaged posterior distribution for ten identically prepared synthetic data sets for the K summary statistic, where $K = \{d[q(i)] + d[N]\}/2$. Synthetic data was generated with $P_m = 0.25$, $P_p = 2 \times 10^{-3}$. The maximum distance between summary statistics for θ to be accepted was (a) $\epsilon = 0.015$, (b) $\epsilon = 0.0135$, (c) $\epsilon = 0.012$. Red indicates high relative frequency while blue indicates low relative frequency.

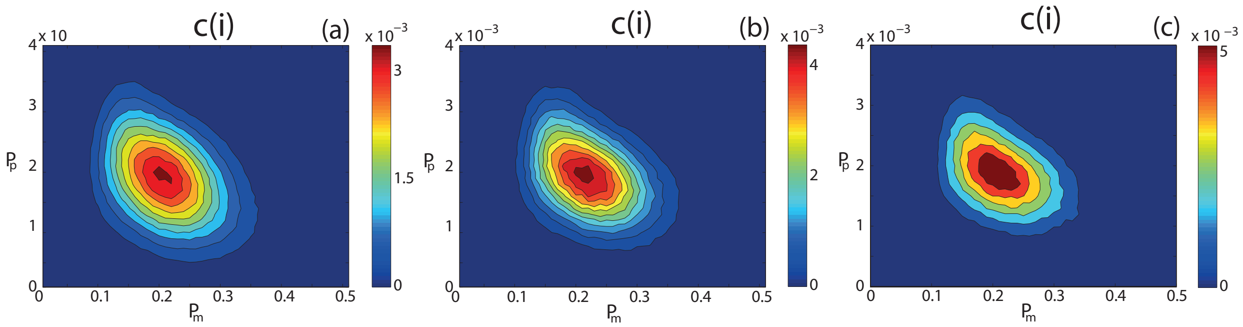

Figure 6: Convergence of the averaged posterior distribution for ten identically prepared synthetic data sets for the summary statistic consisting of the counts of pair distances $c(i)$. Synthetic data was generated with $P_m = 0.25$, $P_p = 2 \times 10^{-3}$. The maximum distance between summary statistics for θ to be accepted was (a) $\epsilon = 0.075$, (b) $\epsilon = 0.065$, (c) $\epsilon = 0.06$. Red indicates high relative frequency while blue indicates low relative frequency.

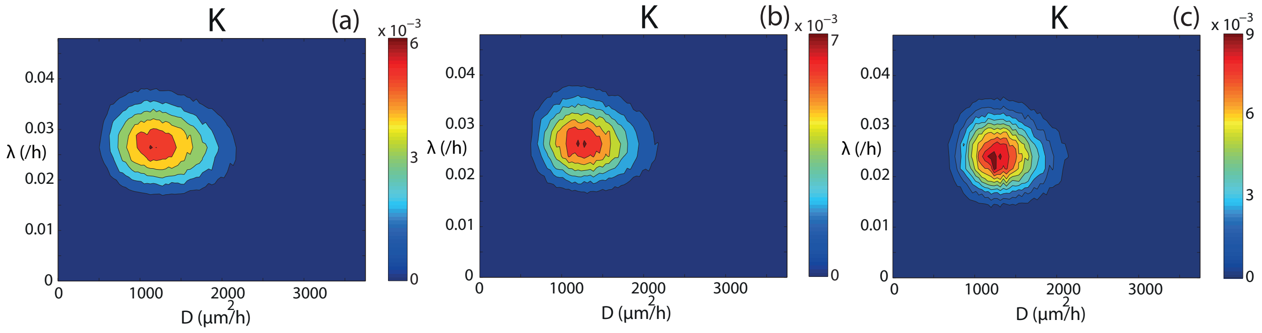

Figure 7: Convergence of the posterior distribution for a single experimental data set generated from a scratch assay using the K summary statistic, where $K = \{d[q(i)] + d[N]\}/2$. Synthetic data was generated with $P_m = 0.25$, $P_p = 2 \times 10^{-3}$. The maximum distance between summary statistics for θ to be accepted was (a) $\epsilon = 0.02$, (b) $\epsilon = 0.0175$, (c) $\epsilon = 0.015$. Red indicates high relative frequency while blue indicates low relative frequency.
